# Pathogenicity of severe fever with thrombocytopenia syndrome virus in mice regulated in type I interferon signaling: Severe fever with thrombocytopenia and type I interferon

**DOI:** 10.1186/s42826-020-00070-0

**Published:** 2020-10-21

**Authors:** Seok-Chan Park, Jun Young Park, Jin Young Choi, Sung-Geun Lee, Seong Kug Eo, Jae-Ku Oem, Dong-Seob Tark, Myungjo You, Do-Hyeon Yu, Joon-Seok Chae, Bumseok Kim

**Affiliations:** 1 College of Veterinary Medicine, Jeonbuk National University, Iksan, 54596 Republic of Korea; 2 Korea Zoonosis Research Institute, Jeonbuk National University, Iksan, 54531 Republic of Korea; 3 College of Veterinary Medicine, Gyeongsang National University, Jinju, 52828 Republic of Korea; 4 Laboratory of Veterinary Internal Medicine, BK21 PLUS Program for Creative Veterinary Science Research, Research Institute for Veterinary Science and College of Veterinary Medicine, Seoul National University, 1 Gwanak-ro, Gwanak-gu, Seoul, 08826 Republic of Korea

**Keywords:** Severe fever with thrombocytopenia syndrome, Type I interferon, Mice, Pathogenicity

## Abstract

Severe fever with thrombocytopenia syndrome (SFTS) is an emerging zoonotic disease, which causes high fever, thrombocytopenia, and death in humans and animals in East Asian countries. The pathogenicity of SFTS virus (SFTSV) remains unclear. We intraperitoneally infected three groups of mice: wild-type (WT), mice treated with blocking anti-type I interferon (IFN)-α receptor antibody (IFNAR Ab), and IFNAR knockout (IFNAR^−/−^) mice, with four doses of SFTSV (KH1, 5 × 10^5^ to 5 × 10^2^ FAID_50_). The WT mice survived all SFTSV infective doses. The IFNAR Ab mice died within 7 days post-infection (dpi) with all doses of SFTSV except that the mice were infected with 5 × 10^2^ FAID_50_ SFTSV. The IFNAR^−/−^ mice died after infection with all doses of SFTSV within four dpi. No SFTSV infection caused hyperthermia in any mice, whereas all the dead mice showed hypothermia and weight loss. In the WT mice, SFTSV RNA was detected in the eyes, oral swabs, urine, and feces at 5 dpi. Similar patterns were observed in the IFNAR Ab and IFNAR^−/−^ mice after 3 dpi, but not in feces. The IFNAR Ab mice showed viral shedding until 7 dpi. The SFTSV RNA loads were higher in organs of the IFNAR^−/−^ mice compared to the other groups. Histopathologically, coagulation necrosis and mononuclear inflammatory cell infiltration in the liver and white pulp atrophy in the spleen were seen as the main lesions in the IFN signaling lacking mice. Immunohistochemically, SFTSV antigens were mainly detected in the marginal zone of the white pulp of the spleen in all groups of mice, but more viral antigens were observed in the spleen of the IFNAR^−/−^ mice. Collectively, the IFN signaling-deficient mice were highly susceptible to SFTSV and more viral burden could be demonstrated in various excreta and organs of the mice when IFN signaling was inhibited.

## Introduction

Severe fever with thrombocytopenia syndrome (SFTS) is an emerging viral zoonotic disease, caused by *Dabie Bandavirus* [former SFTS virus (SFTSV)]. SFTSV is a tick-borne virus belonging to the Genus *Bandavirus* (former *Huaiyangshan Banyangvirus*), Family *Phenuiviridae*. SFTSV causes high fever, thrombocytopenia, and leukocytopenia, followed by multi-organ dysfunction (MOD) and death in humans in East Asian countries [1]. Since SFTSV was first identified in China in 2010, SFTS cases have been identified in China, Japan, South Korea, Vietnam, and Taiwan [2]. The fatality rate of SFTS is high (6–32%) but more specific or effective treatments or vaccines have not been developed yet [3]. Thus, SFTS is listed in the World Health Organization Prioritized Pathogens, which pose major public health risks and further research and development are needed [4].

To study the pathogenesis of SFTSV and make therapeutics and vaccines, animal models with human-like symptoms are essential. At present, rhesus macaque monkeys, which had shown symptoms similar to humans in other viral diseases, did not show human-like symptoms when infected with SFTSV [5]. Old ferrets and cats have been shown to exhibit human-like symptoms when infected with SFTSV, but many immune-related functional studies cannot be performed in these animals. Therefore, they have limitations as animal models compared to mice [6, 7].

Interferons (IFNs) are key effectors of viral infection in innate and adaptive immune systems and are categorized into three groups (type I, II, and III) [8]. Type I IFNs bind to a common IFN-α/β receptor (IFNAR), which initiates a signaling cascade that induces the expression of thousands of antiviral effector function genes. Previous studies showed that type I IFN response is essential for SFTSV infection [9–11]. Several studies have been conducted using mice as animal models of SFTSV. The studies reported that no clinical symptoms or deaths occurred in mice with normal immune responses infected with SFTSV. Only type I interferon (IFN)-deficient mouse models, newborn mice, mitomycin-treated mice, and STAT2-knockout (KO) hamsters are available as models for the fatal illness caused by SFTSV infection [10, 12]. IFNAR KO (IFNAR^−/−^) mice have been previously used as small animal models for SFTSV infection and other emerging viruses, such as Zika virus (ZIKV) and Rift valley fever virus [11, 13–15]. However, IFNAR^−/−^ mice models have particular use because they lack a key component of antiviral immunity.

Thus, we utilized the IFNAR antibody (Mab, MAR1-5A3) to inhibit the IFN signaling temporarily, which allowed the mice with intact IFNAR, a key component of antiviral immunity, to be infected with SFTSV.

## Materials and methods

### Viruses and cells

The Korean SFTSV strain KH1 (GenBank accession no. MH491547, MH491548.1, MH491549.1) was kindly provided by Dr. Joon-Seok Chae [16]. This virus was passaged five times on monolayers of Vero E6 cells in DMEM containing 5% fetal bovine serum (FBS) with antibiotics (penicillin; 100 U/ml, streptomycin; 100 μg/ml). The virus infectivity titers were determined through a fluorescence active infectious dose (FAID_50_) assay.

### Animal infection and sample collection

Six-week-old female C57BL/6 mice (WT, Samtako, Kyoung Gi-do, Korea) and 6-week-old type 1 IFN receptor knockout (IFNAR^−/−^) female mice were used in this study. To study the correlation between immunologic competence and the pathogenicity of SFTSV, MAR1-5A3 (mouse anti-mouse IFNAR, IgG1) monoclonal antibody (MAb) was administered as an intraperitoneal (IP) injection of 100 μg per mouse 1 day before SFTSV infection and 2 days post-infection (dpi) to the C57BL/6 mice (IFNAR Ab mice). The IFNAR^−/−^ mice, purchased from B&K Universal (Hull, UK), were backcrossed onto C57BL/6 mice for at least 10 generations. The Mice were inoculated intraperitoneally (i.p.) with 5 × 10^5^ to 5 × 10^2^ FAID_50_ in a volume of 200 μL of phosphate-buffered saline (PBS). Body temperature was obtained using the rectal probes. Blood samples were collected from the retro-orbital venous plexus and analyzed with an automatic blood cell counter (Exigo-Vet., Boule Medical AB Inc., Stockholm, Sweden). The sera were frozen at − 80 °C until further analysis. Eye and oral swabs, feces, and urine samples were placed in 200 μL of PBS and stored at − 80 °C. The SFTSV infected mice were euthanized and the organs were harvested aseptically. All SFTSV infection studies were performed in an animal biosafety level 3 laboratory. All animal experiments followed the requirements of the Animal Care and Ethics Committees of Jeonbuk National University (approval number: JBNU 2019–002).

### Detection of SFTSV RNA by reverse transcription-polymerase chain reaction

Total RNA was isolated by using Hybrid-R™ (GeneAll, Seoul, Korea). The RNA was reverse transcribed using a ReverTra Ace qPCR RT Master Mix (TOYOBO, Osaka, Japan) to generate cDNA. The viral copy numbers were determined by real-time PCR (RT-PCR) with an L segment-based SFTSV-specific primer. The forward primer sequence was SFTSV-L-F: AACATCCTGGACCTTGCATC and the reverse primer sequence was SFTSV-L-R: CAATGTGGCCATCTTCTCCA [17]. Copy numbers were determined by comparison to a standard control. RT-PCR was performed using CFX96 Real-time PCR (Bio-Rad Laboratories, Hercules, CA) with qPCR SyGreen Mix (PCRbiosystems, Seoul, Korea).

### Histopathology and immunohistochemistry

In the 5 × 10^2^ FAID_50_ SFTSV infection experiment, four mice from each group were necropsied 1, 3, 7, 12, and 17 dpi, and samples of the brain, lung, liver, spleen, kidney, small intestine (s. intestine), and large intestine (l. intestine) were obtained for histopathologic and immunohistochemical examination and SFTSV genome quantification by RT-PCR. The collected organ tissues were fixed with 10% neutral phosphate-buffered formalin and processed and embedded in paraffin according to standard procedures. The embedded tissues were sectioned to 6 μm thicknesses using a microtome (HM-340E; Thermo Fisher Scientific Inc., Waltham, MA, USA) and placed on slides. The sectioned tissues were stained with hematoxylin and eosin (H&E) according to a standard protocol. To detect the viral antigen by immunohistochemistry, a mouse monoclonal antibody against SFTSV N protein (kindly provided by Dr. Jun-Gu Choi, Animal and Plant Quarantine Agency, Korea) was used as the primary antibody.

### Statistical analysis

Unless otherwise indicated, comparisons between the groups in the in vivo experiments were performed by a 2-tailed Mann-Whitney test or one-way ANOVA followed by Tukey’s multiple comparison test. A *p*-value of < 0.05 was considered statistically significant. Statistical analyses were performed by GraphPad Prism version 8.00.

## Results

### Susceptibility to SFTSV in mice deficient in type I interferon signaling

**Fig. 1:**
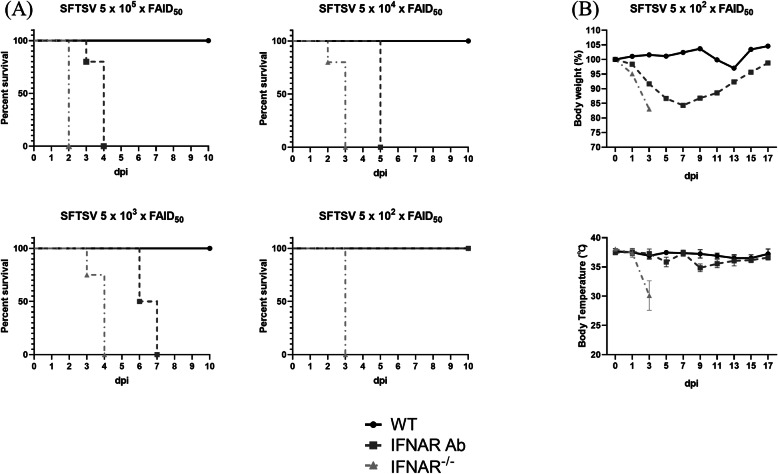
Survival rate, weight loss, and body temperature of mice with different type I interferon signaling after SFTSV challenge. Three groups of mice, C57 BL/6 (WT), mice treated with blocking anti-type I interferon-α receptor antibody (IFNAR Ab), and IFNAR knockout (IFNAR^−^/^−^) mice, were intraperitoneally infected with four doses of SFTSV (KH1, 5 × 10^5^ -5 × 10^2^ FAID_50_). **a** Survival rates, **b** body weight (%), and body temperature (°C) were analyzed

Three groups of mice were intraperitoneally infected (WT, IFNAR Ab, and IFNAR^−/−^ mice) with four doses of SFTSV (KH1, 5 × 10^5^ to 5 × 10^2^ FAID_50_). The WT mice showed no mortality from any dose of SFTSV (Fig. 1a). In mice infected with 5 × 10^5^, 5 × 10^4^, and 5 × 10^3^ FAID_50_ SFTSV, the IFNAR Ab mice showed 100% mortality 4, 5, and 7 dpi, respectively, and showed steady weight loss until just before death. However, in the 5 × 10^2^ FAID_50_ SFTSV infection experiment, the IFNAR Ab mice did not succumb to SFTSV and recovered the lost weight after 7 dpi (Fig. 1b). This finding in the IFNAR Ab mice given a low dose SFTSV infection might be due to the half-life of IFNAR Ab, which is 5.2 days. The antibody was injected 1 day before infection and 2 dpi [18]. The IFNAR^−/−^ mice succumbed to SFTSV regardless of the SFTSV dose (5 × 10^5^ to 5 × 10^2^ FAID_50_) within 4 dpi. No SFTSV-infected mice had hyperthermia (Fig. 1b), whereas all the dead mice showed temperature reduction, ruffled fur, anorexia, depression, gastrointestinal symptoms, and loss of weight after virus infection. To study the pathogenesis of SFTSV and the potential role of IFNAR Ab mice as an animal model for SFTSV, 20 mice of each group were inoculated with 5 × 10^2^ FAID_50_ SFTSV. The hematologic changes were observed and viral burden determined.

### Hematologic parameters

**Fig. 2:**
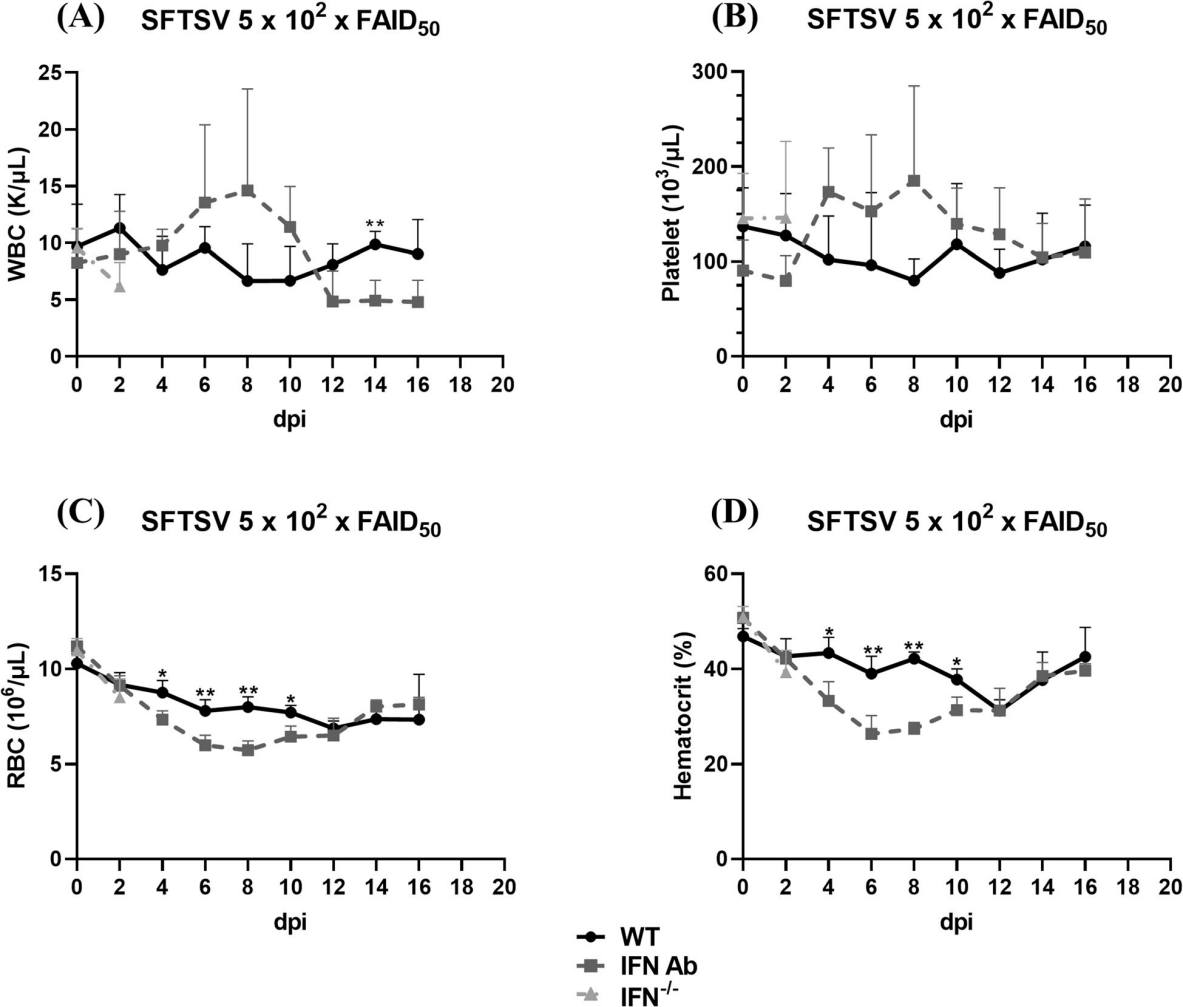
Blood cell count. Complete blood cell counts were conducted using an automated blood cell counter. The WBCs (**a**), platelets (**b**), RBCs (**c**), and hematocrit (HCT) (**d**) were analyzed. The values shown are the mean + SD from five mice per group. * *p* < 0.05, **p < 0.01 relative to WT mice, Mann-Whitney test

To evaluate the hematologic changes in the SFTSV-infected mice, blood samples were collected by retro-orbital plexus puncture every 2 days and complete blood counts were performed. White blood cell (WBC) counts in the WT mice remained within the reference range (2600–10,100/μL), but the WBC count in the IFNAR Ab mice increased slightly from 4 to 10 dpi and recovered after 12 dpi (Fig. 2a). Severe thrombocytopenia was not detected in any mice after SFTSV infection (Fig. 2b). A decreasing pattern of RBCs and hematocrit levels was observed in both groups until 12 dpi and the levels were significantly lower in the IFNAR Ab mice compared to the WT mice from 4 to 10 dpi (Fig. 2c, d).

### SFTS viral load in various organs

**Fig. 3:**
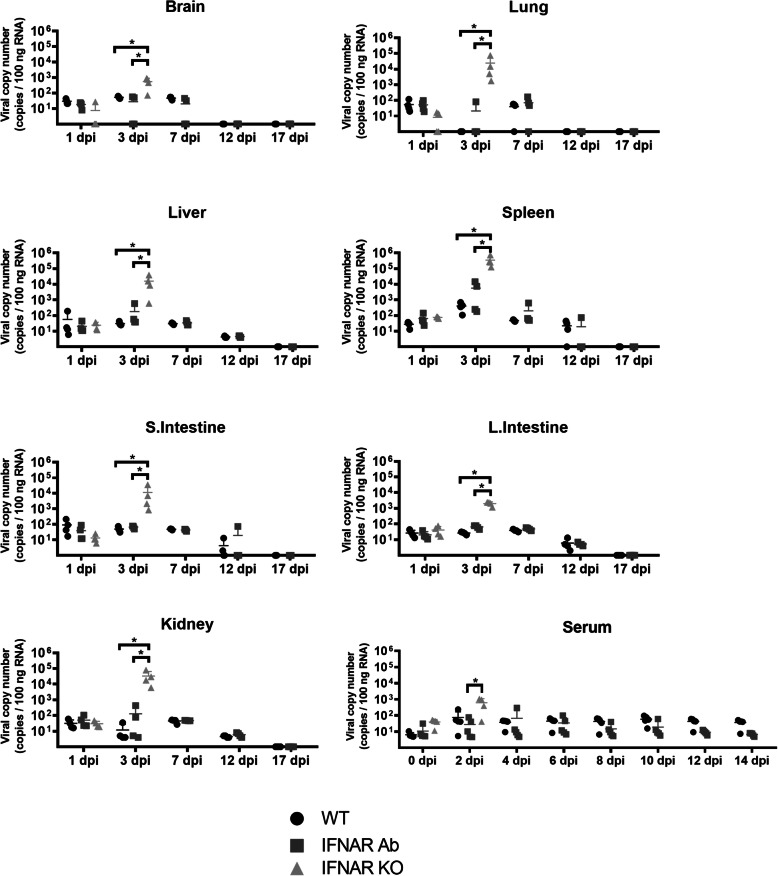
SFTS viral qPCR results. Organs were harvested from WT and IFNAR Ab mice 1, 3, 7, 12, and 17 dpi with 5 × 10^2^ FAID_50_ SFTSV by i.p. infection. Organs from the IFNAR^−/−^ mice were collected 1 and 3 dpi. Serum samples were collected 0, 2, 4, 6, 8, 10, 12, and 14 dpi. The solid lines indicate the mean + SD from four mice per group. The SFTSV RNA loads were higher in the brain, lung, liver, spleen, small intestine, large intestine, kidney, and serum of the mice. * *p* < 0.05, relative to IFNAR^−/−^ mice, Mann-Whitney test

To study the pathogenesis of SFTSV, four mice of each 5 × 10^2^ SFTSV-infected group were necropsied 1, 3, 7, 12, and 17 dpi and the viral load for each organ were evaluated. The SFTSV RNA in the tissues, including the brain, lung, liver, spleen, kidney, s. intestine, and l. intestine, was analyzed by RT-PCR. At 1 dpi, SFTSV RNA was detected in all the tested organs, but there was no significant difference in viral copy number among the groups of mice (Fig. 3). However, 3 dpi, the IFNAR^−/−^ mice showed significantly higher (*p* < 0.05) SFTSV RNA levels in all organs than those of the other two groups of mice. The highest SFTSV RNA levels were detected in the spleen compared to the other organs of the IFNAR^−/−^ mice. Similar SFTSV RNA viral levels were detected in all organs of the WT and IFNAR Ab mice. SFTSV RNA was not detected after 12 dpi in the brain and lung, and after 17 dpi in the liver, kidney, s. intestine, and l. intestine of the WT and IFNAR Ab mice. In the serum samples, the IFNAR^−/−^ mice showed significantly higher SFTSV RNA than that of the IFNAR Ab mice 2 dpi. SFTSV RNA was detected consistently until 14 dpi in the WT and IFNAR Ab mice.

### SFTS viral RNA detection in mice excreta

In mice infected with 5 × 10^2^ FAID_50_ SFTSV, eye and oral swabs, and urine and feces were collected 1, 3, 5, 7, 9, 11, 13, 15, and 17 dpi to evaluate viral antigen shedding and the viral load for each sample were evaluated. The WT mice showed no clinical symptoms at any day post-infection with SFTSV. However, SFTS viral RNA was detected in the eye and oral swabs, and urine and feces 5 dpi (Fig. 6). In the case of the IFNAR Ab and IFNAR^−/−^ mice, SFTSV RNA was detected earlier than that of the WT mice in the eye and oral swabs, and urine (after 3 dpi), and the IFNAR Ab mice showed longer viral shedding than the WT mice (up to 7 dpi). These results showed that the SFTSV-infected mice could shed SFTS viral RNA, regardless of clinical symptoms, and SFTS viral RNA could be released for a longer time in mice with downregulated IFN signaling.

### Pathological examination

**Fig. 4:**
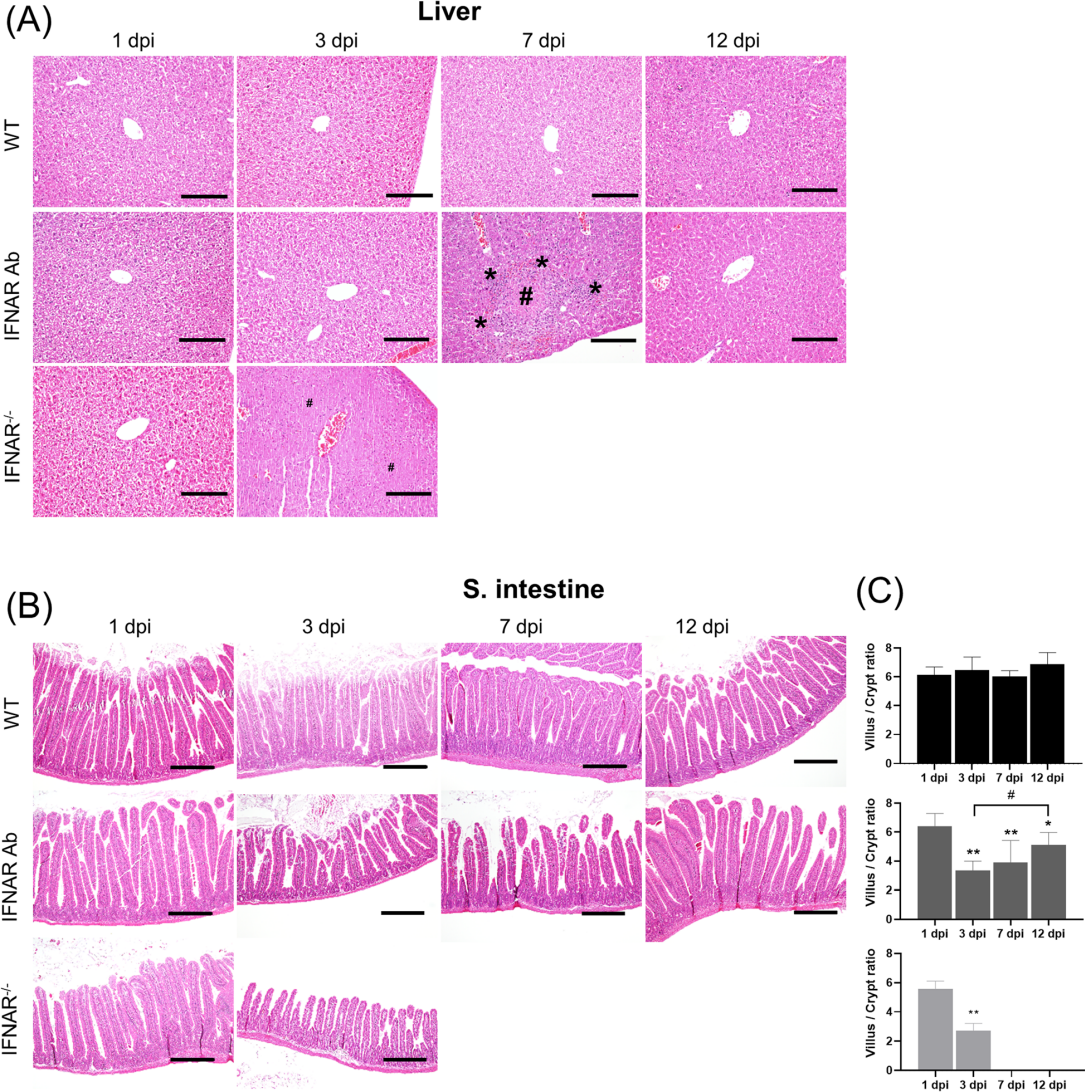
Histopathological examinations and villus/crypt ratio from the SFTSV-infected mice. Mice were inoculated i.p. with 5 × 10^2^ FAID_50_ SFTSV. The liver and small intestine of the C57 BL6 wild-type (WT), mice treated with blocking anti-type I interferon (IFN)-α receptor antibody (IFNAR Ab) were collected 1, 3, 7, and 12 dpi and the organs of the IFNAR knockout (IFNAR^−/−^) mice were collected 1 and 3 dpi after infection. **a** The crosshatches and asterisks indicate coagulation necrosis and mononuclear cell infiltration in the liver, respectively. Bars 100 μm. **b** The WT mice had long, intact, regular villi at all time points, but the IFNAR Ab and IFNAR^−/−^ mice showed shortened, damaged, blunting villi, and the IFNAR Ab mice recovered at 12 dpi. Bars 200 μm. **c** At least 20 typical villi and crypts were measured for each mouse. The villus/crypt ratios are expressed as the mean ± standard deviation. Statistical analyses were performed using one-way ANOVA followed by Tukey’s multiple comparison test. *p < 0.05 and **p < 0.01 for 1 dpi vs. other dpi for the same group mice. #*p* < 0.05 for 3dpi vs. 12 dpi for the IFNAR AB mice

**Fig. 5:**
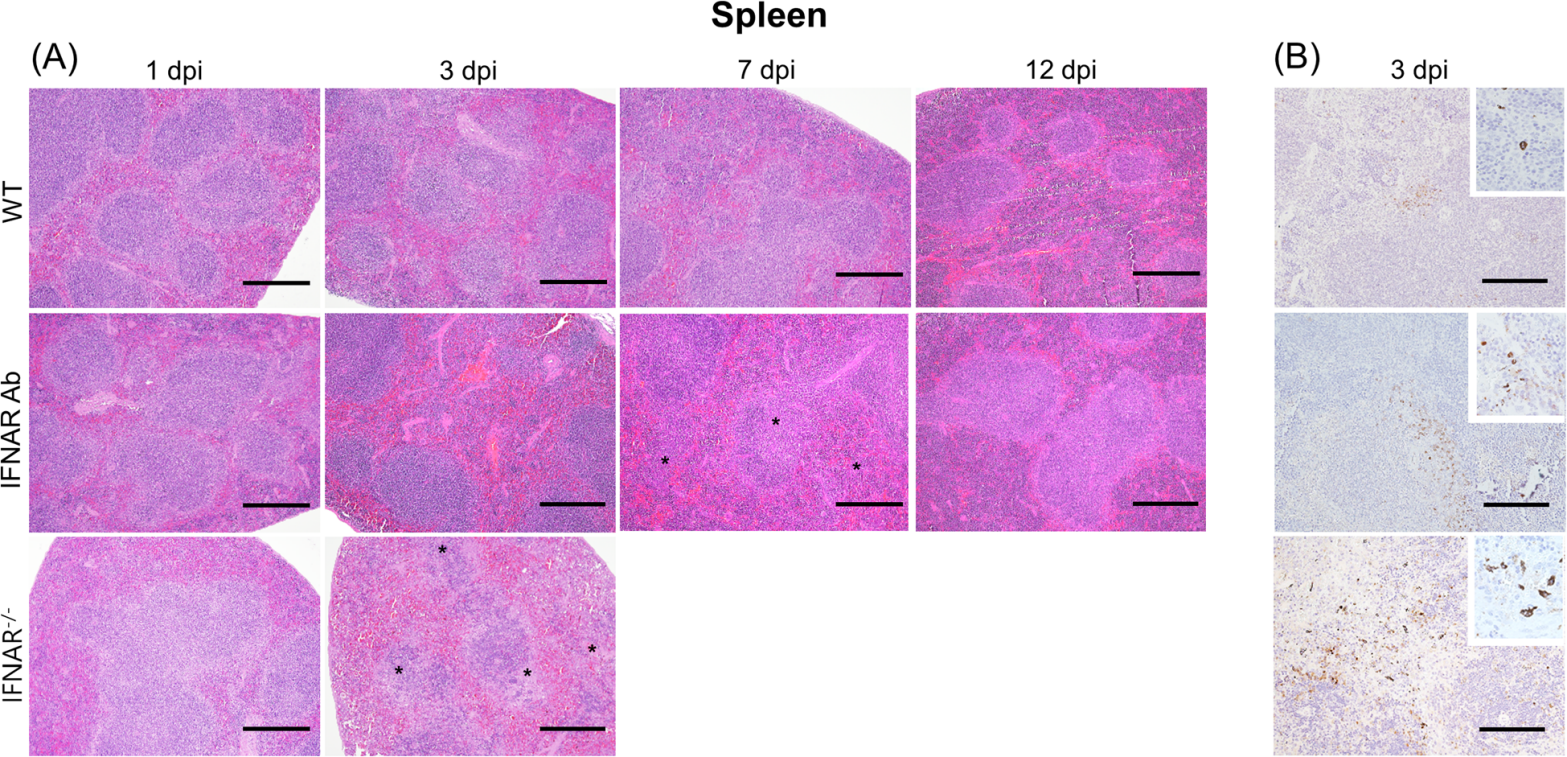
Histopathological and immunohistochemical examinations of the spleen collected from the SFTSV-infected mice. Mice were inoculated i.p. with 5 × 10^2^ FAID_50_ SFTSV. Spleens of the WT and IFNAR Ab mice were collected 1, 3, 7, 12 dpi and the spleens of the IFNAR^−/−^ mice were collected 1 and 3 dpi. **a** Asterisks indicate white pulp atrophy. Bars, 200 μm. H&E, hematoxylin and eosin staining. **b** The SFTSV N protein antigens were confirmed in the spleens by immunohistochemistry (IHC). Bars, 100 μm

To assess the progression of SFTSV over time, histopathology and IHC in each organ was examined in the necropsy tissues of four mice inoculated i.p with 5 × 10^2^ FAID_50_ SFTSV at 1, 3, 7, 12, and 17 dpi. The WT mice had no observable histological abnormalities. Between the two IFN signaling-regulated mice groups, lesions were observed sooner in the IFNAR^−/−^ mice. At 3 dpi, coagulation necrosis in the liver and white pulp atrophy, especially in the marginal zone of the spleen of the IFNAR^−/−^ mice, were observed (Figs. 4a and 5a). At 7 dpi, coagulation necrosis and mononuclear inflammatory cell infiltration around the necrotic lesions of the liver and white pulp atrophy in the spleen of the IFNAR Ab mice were observed. Since one of the major clinical manifestations of SFTS is gastrointestinal symptoms and the SFTSV-infected IFNAR Ab and IFNAR^−/−^ mice showed gastrointestinal symptoms, we histopathologically evaluated the s. intestine and measured the villus/crypt depth (V/C) ratio in the s. intestine. At least 20 V/C ratios were measured for each mouse in the proximal s. intestine. The WT mice had long, intact, regular villi at all time points, and the V/C ratios did not differ significantly (Fig. 4b, c). However, the IFNAR Ab and IFNAR^−/−^ mice showed shortening, damaged, blunting villi, and the V/C ratios significantly decreased from 3 dpi. All IFNAR^−/−^ mice succumbed to SFTSV at 3 dpi, but the surviving IFNAR Ab mice recovered villus morphology and V/C ratio from 12 dpi comparable to 1 dpi. In the present study, SFTS viral RNA was the most enriched in the spleen of all SFTSV-infected mice and previous studies suggested that the spleen may be the primary target organ of SFTSV [10, 19]. Therefore, we performed immunohistochemistry assays with spleen sections isolated 3 dpi using a monoclonal antibody against SFTSV N protein (NP). As shown in Fig. 5b, the frequency of SFTSV NP antigen-positive cells was in the order of IFNAR^−/−^, IFNAR Ab, and WT mice, consistent with the qPCR results of this study. SFTSV NP antigen-positive cells were rarely detected in the white pulp but mainly detected in the marginal zone of the spleen, and were monocyte-like cells in all groups of mice.

## Discussion

In this study, we intraperitoneally inoculated different titers of SFTSV into WT, IFNAR Ab, and IFNAR^−/−^ mice. Similar to previous studies, the no immunocompetent WT mice showed any clinical signs or died. However, the IFNAR^−/−^ mice succumbed to SFTSV within 3 dpi even at small titers of SFTSV (5 × 10^2^ FAID_50_). Therefore, the IFNAR^−/−^ mice might be not suitable to represent the clinical symptoms of human SFTSV infection. The IFNAR Ab mice showed similar survival rates after infection following inoculation with 5 × 10^5^ to 5 × 10^3^ FAID_50_. However, in mice infected with 5 × 10^2^ FAID_50_ SFTSV, the IFNAR Ab mice survived and recovered from 7 dpi (Fig. 1a). These findings showed that immunocompetent mice could be infected with SFTSV by partially blocking type I IFN signaling. A previous study tried to make a mouse model that did not cause clinical symptoms in immunocompetent mice by injections of type 1 IFNAR Ab to mice infected with ZIKV [14]. The main symptom of ZIKV is related to central nervous system effects. However, the type I IFNAR Ab did not pass through the blood-brain barrier (BBB), so the main symptoms of ZIKV infection were not recapitulated. But, in the present study, the IFNAR Ab mice recapitulated the fatality of SFTSV infections because the main target of SFTSV is a parenchymal organ, such as the spleen. These findings suggest that transgenic mice can be used for SFTS studies after the administration of IFNAR Ab, allowing the study of various pathways involved in SFTSV infections. At present, the strongest candidate animal model of SFTSV infection might be aged ferrets because they exhibit symptoms similar to humans. However, because aged ferrets are outbred and generally unavailable for commercial use, this model has limitations in studying the pathogenesis of SFTS [6].

**Fig. 6:**
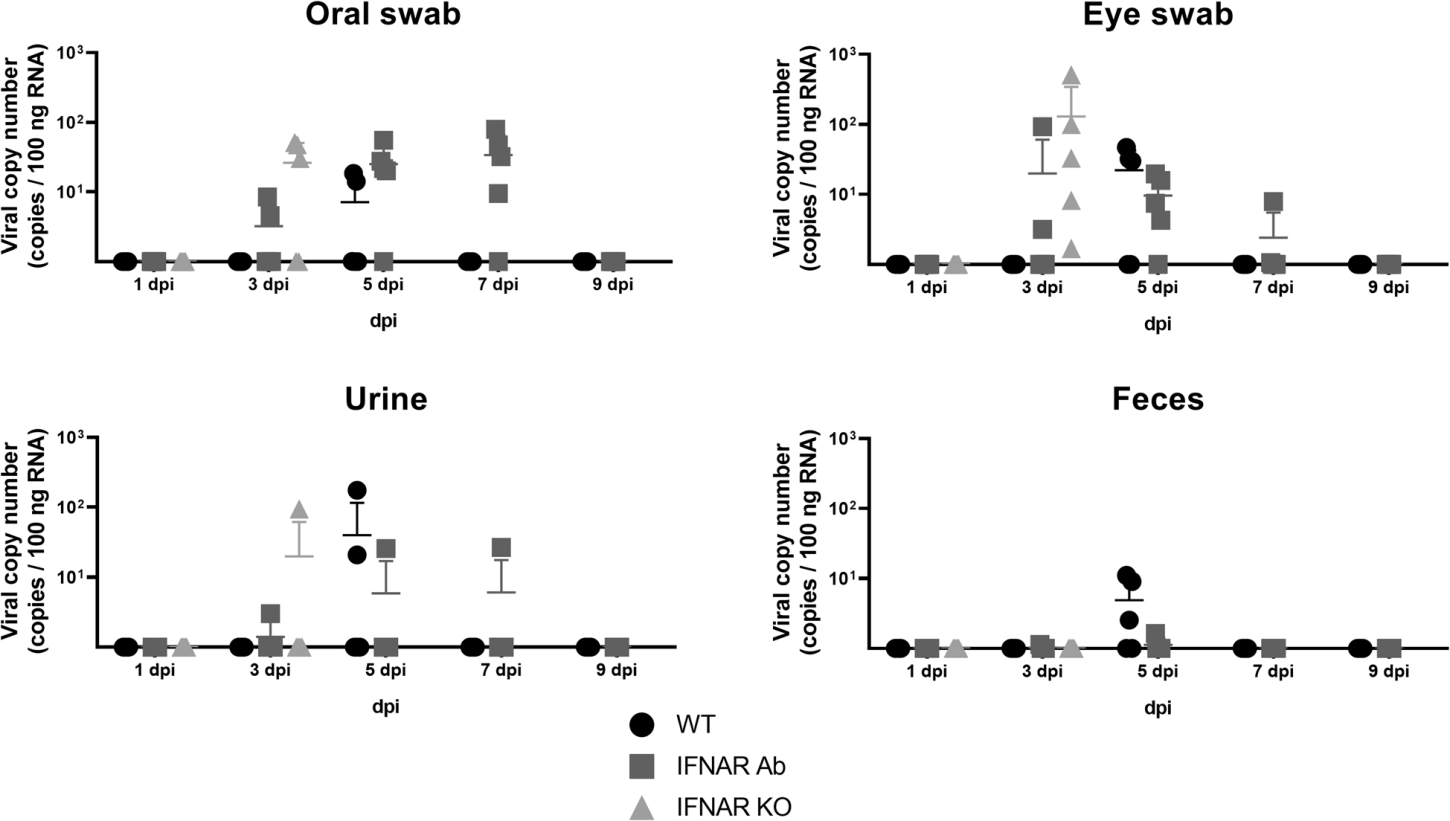
Viral load detected in oral, eye swabs, urine and feces obtained from SFTSV-infected mice. Mice were inoculated i.p. with 5 × 10^2^ FAID_50_ SFTSV. Samples of WT and IFNAR Ab mice were collected 1, 3, 5, 7 and 9 dpi and the samples of IFNAR KO mice were collected 1, 3 dpi. The solid lines indicate the mean + SD from five mice per group

In public health, the risk of infection from asymptomatic carriers has increased [20, 21]. Although human-to-human or cat-to-human SFTSV transmissions have been reported, the risk of infection from an asymptomatic animal carrier of SFTSV has not been confirmed [22, 23]. In this study, we confirmed that immunocompetent, asymptomatic WT mice shed SFTS viral RNA 5 dpi through various excreta (Fig. 6). As expected, the IFNAR Ab mice, which showed clinical signs, shed SFTS viral RNA from 3 to 7 dpi. Since our system could only detect and measure SFTSV RNA, the infectious titers of SFTSV need to be determined in a future study. Furthermore, studies on whether asymptomatic carrier mice can infect other mice are needed.

A previous study suggested that SFTSV targeted macrophages, immature B cells, and reticular cells in the IFNAR^−/−^ mice [10]. The IHC results showed that SFTSV antigens were mainly detected in monocyte-like cells in the marginal zone of the white pulp of spleen, regardless of the group of mice (Fig. 5b). Marked marginal zone reduction in the spleen was the main difference between the dead IFNAR^−/−^ and the surviving mice (Fig. 5a). The marginal zone has a key role in the antiviral response and especially, marginal zone metallophilic macrophages (MOMA) are important for initiating an antiviral B cell response [24]. Furthermore, deficiency in humoral responses and the disruption of B cell immunity affected fatal human SFTSV infection [25]. Based on these results, it is speculated that reductions in the marginal zone and MOMA might affect humoral response deficiency in the SFTSV-infected mice without IFN signaling. Therefore, it will be necessary to determine the mechanism of how IFN signaling deficiency affects humoral immunity after SFTSV infection.

Our findings showed that healthy asymptomatic mice could shed SFTSV via various excreta and the IFNAR Ab mice had some limitations in hematologic manifestation but recapitulated the fatality of SFTSV infections.

## Conclusions

Regulation of type I IFN signaling by injecting type 1 IFNAR Ab into different types of immunocompromised mice will help to elucidate the pathogenesis of SFTSV infection with small quantities of virus.

## Data Availability

Not applicable.

## Abbreviations

SFTS: Severe fever with thrombocytopenia syndrome
SFTSV: SFTS virus
WT: Wild-type
IFN: Interferon
IFNAR Ab: IFN-α receptor antibody
IFNAR^−/−^: IFNAR knockout
dpi: Days post-infection
ZIKV: Zika virus
FBS: Fetal bovine serum
FAID_50_: Fluorescence active infectious dose
MAb: Monoclonal antibody
PBS: Phosphate-buffered saline
RT-PCR: Real-time PCR
s. intestine: Small intestine
l. intestine: Large intestine
H&E: Hematoxylin and eosin
WBC: White blood cell
V/C ratio: Villus/crypt depth
NP: N protein
BBB: Blood-brain barrier
MOMA: Marginal zone metallophilic macrophages
